# Viruses and Autoimmunity: A Review on the Potential Interaction and Molecular Mechanisms

**DOI:** 10.3390/v11080762

**Published:** 2019-08-19

**Authors:** Maria K. Smatti, Farhan S. Cyprian, Gheyath K. Nasrallah, Asmaa A. Al Thani, Ruba O. Almishal, Hadi M. Yassine

**Affiliations:** 1 Biomedical Research Center, Qatar University, Doha 2713, Qatar; 2 Basic Medical Science Department, College of Medicine-QU Health, Qatar University, Doha 2713, Qatar; 3 Department of Biomedical Sciences, College of Health Science-QU Health, Qatar University, Doha 2713, Qatar

**Keywords:** autoimmunity, viral infections, molecular mechanisms, molecular mimicry

## Abstract

For a long time, viruses have been shown to modify the clinical picture of several autoimmune diseases, including type 1 diabetes (T1D), systemic lupus erythematosus (SLE), rheumatoid arthritis (RA), Sjögren’s syndrome (SS), herpetic stromal keratitis (HSK), celiac disease (CD), and multiple sclerosis (MS). Best examples of viral infections that have been proposed to modulate the induction and development of autoimmune diseases are the infections with enteric viruses such as Coxsackie B virus (CVB) and rotavirus, as well as influenza A viruses (IAV), and herpesviruses. Other viruses that have been studied in this context include, measles, mumps, and rubella. Epidemiological studies in humans and experimental studies in animal have shown that viral infections can induce or protect from autoimmunopathologies depending on several factors including genetic background, host-elicited immune responses, type of virus strain, viral load, and the onset time of infection. Still, data delineating the clear mechanistic interaction between the virus and the immune system to induce autoreactivity are scarce. Available data indicate that viral-induced autoimmunity can be activated through multiple mechanisms including molecular mimicry, epitope spreading, bystander activation, and immortalization of infected B cells. Contrarily, the protective effects can be achieved via regulatory immune responses which lead to the suppression of autoimmune phenomena. Therefore, a better understanding of the immune-related molecular processes in virus-induced autoimmunity is warranted. Here we provide an overview of the current understanding of viral-induced autoimmunity and the mechanisms that are associated with this phenomenon.

## 1. Introduction

**Figure 1:**
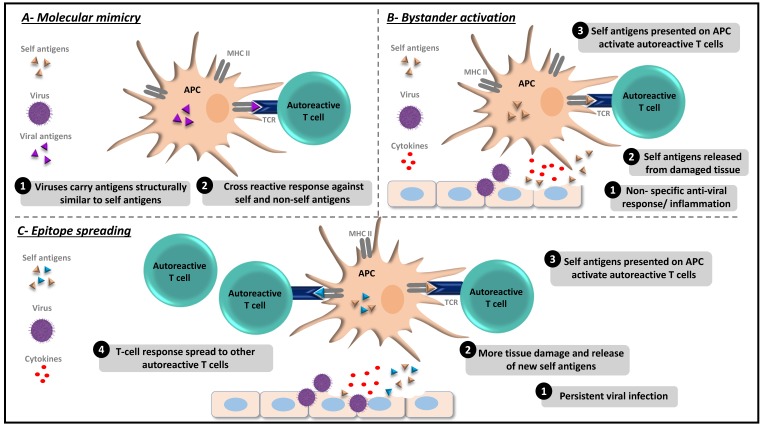
Mechanisms of virus-induced autoimmunity. (**A**) Molecular mimicry model: (1) Viruses carry epitopes structurally similar to self-epitopes. (2) Presentation of viral epitopes by antigen presenting cells (APCs) activate autoreactive T cells that bind to both, self and non-self-antigens, and induce tissue damage. (**B**) Bystander activation model: (1) Non-specific and over reactive antiviral immune responses lead to the liberation of self-antigens and release of inflammatory cytokines from the damaged tissue. (2) Self-antigen is taken up and presented by APCs. (3) Autoreactive T cells activated by APCs, leading to tissue destruction. (**C)** Epitope spreading model: (1) Persistent viral infection. (2) Continued tissue damage and release of new self-antigens. (3) Self-antigens are taken up and presented by APCs. (4) Nonspecific activation of more autoreactive T cells leading to autoimmunity.

Autoimmune diseases (AID) develop as a result of an aberrant immune response in recognizing self and non-self-antigens. Currently, there are more than 80 identified types of autoimmune disorders [1]. Although the etiologies of several autoimmune disorders remain completely understood, multiple factors have been linked to autoimmune responses, including genetics, age, environment, as well as viral infections. Viruses have been considered as major environmental factors that trigger the autoimmune phenomena in genetically susceptible individuals [2,3]. Multiple mechanisms have been proposed to explain the breakdown of self-tolerance by viral infections (Figure 1). Traditionally, it was believed that viruses carry structurally similar antigens to self-antigens, which activate B and T cells and lead to a cross-reactive response against both self- and non-self-antigens, a mechanism known as “molecular mimicry” [4]. Molecular mimicry has been described for herpes simplex virus (HSV)-induced stromal keratitis [5], virus-induced diabetes [6], autoimmune myocarditis that is mediated by Coxsackie virus infection [7], Theiler’s murine encephalomyelitis virus-induced demyelinating disease (TMEV-IDD) [8], and several others [9]. Another hypothesized mechanism is the “bystander activation”, whereby a non-specific and an over-reactive antiviral immune response creates a localized pro-inflammatory environment along with the release of self-antigens from the damaged tissue. These self-antigens are subsequently taken up and presented by antigen presenting cells (APC) to stimulate the previously non-responsive, yet autoreactive T cells in the vicinity triggering autoimmunity [10]. A related mechanism is called “epitope spreading”, in which a viral infection triggers the release of more self-antigens and the de novo activation of autoreactive cells, which consequently spread to target additional self-epitopes [9]. Both molecular mimicry and bystander activation have been observed in the experimental autoimmune encephalomyelitis (EAE) model of multiple sclerosis (MS) [11], West Nile virus (WNV)-mediated myasthenia gravis (MG) [12], TMEV-IDD [13], and other disorders [9]. Moreover, viruses may immortalize autoreactive effector cells as observed in Epstein–Barr virus (EBV)-infected B cells [14]. While several hypotheses have been proposed to understand the mechanisms underlying virus-induced autoimmunity, the precise contribution of these mechanisms are not yet fully understood. This review summarizes the recent findings on virally induced autoimmunity and the potential underlying mechanisms triggering the development of this disorder.

## 2. Viral Infections and Induction of Autoimmunity

### 2.1. Enteric Infections 

Enteroviruses are considered the main viral candidates for causing type 1 diabetes in humans [15]. Seasonal incidence of type 1 diabetes has been reported subsequent to enterovirus infections since 1969 [16,17]. Although the role of enteroviruses in type 1 diabetes has been investigated for more than 40 years, the etiological link is still enigmatic [18]. A higher frequency of enterovirus infections has been reported among siblings who develop type 1 diabetes as compared to nondiabetic controls [19]. Additionally, higher titers of enterovirus antibodies were found in pregnant mothers whose children later developed type 1 diabetes [19]. Furthermore, association between enteroviral infections and beta cell autoimmunity has been reported on several occasions. For example, in type 1 diabetes-genetically susceptible children, a seasonal detection of autoantibodies matched the seasonal occurrence of enteroviruses infections, suggesting the contribution of enteroviruses to autoimmune diabetes [20]. In another study, signs of enterovirus infections were detected in parallel with the first appearance of diabetes-associated autoantibodies [21]. Correspondingly, Elshebani et al. found that certain enterovirus strains that are isolated from type 1 diabetes patients affected the function of beta cells, and induced cell destruction in vitro [22]. Similarly, enteroviruses have been found in intestinal biopsies from diabetic patients, suggesting the possibility of an ongoing persistent enterovirus infection in the gut mucosa of type 1 diabetes patients [23]. Furthermore, a recent systematic review that analyzed 24 published papers documented a significant association between enteroviruses and autoimmunity/type 1 diabetes [24]. 

#### 2.1.1. Coxsackie B Viruses

Coxsackie B virus (CVB) is the most prevalent enterovirus in pre-diabetic and diabetic patients [15]. Several studies have reported the presence of CVB RNA in the blood of type 1 diabetes patients [25,26,27,28]. Juhela et al. found that T cell response to CVB4 (serotypes 4 in Coxsackievirus group B) is enhanced in children with type 1 diabetes, and this might be as a result of enterovirus-specific T cell being trapped in the pancreas [29]. In addition, a post-mortem examination of a deceased diabetic child revealed the death of beta cells and the presence of a lymphocytic infiltrate in the islets of Langerhans [30]. In further support of this finding, CVB4 was propagated in mouse pancreatic cells, and when those cells were inoculated into susceptible mice, they developed autoimmune diabetes [30]. Dotta et al. detected CVB4 in pancreatic tissue from three out of six patients with type 1 diabetes. In fact, the virus was able to infect beta cells originating from non-diabetic donors, leading to beta cell functional impairment and strong Natural Killer (NK) cells-mediated inflammation [31]. This observation could be attributed to the high expression of type-I interferon (IFN-1) in beta cells following CVB infection, which leads to rapid beta cell death [32]. Evidence of inflammation and direct cytolysis of beta cells has been shown to produce autoantigens that initiate autoimmunity in turn [15]. Importantly, age seems to play a significant role in the development of virus-induced diabetes. In this regard, infection with CVB4 has been shown to enhance type 1 diabetes in eight-week-old NOD mice but not in a younger age group, suggesting that CVB4 infection can contribute to diabetes progression, only if a threshold level of pre-existing autoreactive T cells were accumulated in the pancreatic islets [33]. Virus-induced inflammatory cytokines are also thought to play an important role in the induction of autoimmunity in a time-dependent manner. For example, expression of Tumor necrosis factor alpha (TNF-α) in the pancreatic islets has been shown to enhance or abrogate autoimmune diabetes depending on the time of expression [34]. 

The molecular basis of autoimmunity in CVB4 infection is proposed to be a molecular mimicry [35], where the 2C non-structural CVB protein has a shared sequence with the glutamic acid decarboxylase 65 enzyme (GAD65), which is predominantly expressed in pancreatic beta cells [36]. This result aligns with previous studies that have investigated the immunological cross-reactivity caused by sequence similarity between self- and non-self-antigens. Particularly GAD65 was found to play an important role in the pathogenesis of type I diabetes as a target autoantigen [37], where the ensuing immune response to GAD65 has been detected before the onset of clinical diabetes [35]. In this regard, it is of note that T cells isolated from type 1 diabetes patients were shown to react with both GAD65 and 2C protein, however, other studies have demonstrated that healthy control groups can also have reactive T cells to GAD65 [38]. This immunological cross-reactivity may not provide the initiative event for triggering diabetes, but can act as an enhancer for disease development [15]. 

Bystander activation was also a suggested mechanism by which CVB4 induce or accelerate diabetes. Mouse studies have indicated that by evoking beta cells damage, CVB4 infection promotes the release of self-antigens, which are then presented by macrophages to autoreactive T cells [32]. 

On the contrary, some studies have not found convincing evidence supporting the CVB-induced diabetes hypothesis [39]. Notably, Sarmineto et al. showed that although an enterovirus epidemic occurred in Cuban population between 2000 and 2001, diabetes rate in the population was not affected [18]. On the other hand, other studies have demonstrated that infection with CVB during the first year of life can have a protective effect against type 1 diabetes [29]. This protection was attributed to the developmental stage of the adaptive immune system where viral infections trigger specific immune responses, including activation of T regulatory (Treg) cells and induction of suppressors which collectively hinder autoimmunity [15]. Furthermore, CVB infection was found to be protective through enhancing Treg cells in individuals who are genetically susceptible to type 1 diabetes but not insulitis, therefore inhibiting islet-specific T cell autoimmunity [17]. However, it is yet to be determined how often CVB can induce autoimmunity, and what are the factors that contribute to beta cells destruction and diabetes development or protection [15]. 

Taken together, molecular mimics of islet autoantigens have been strongly suggested as a factor for CVB-induced autoimmunity. However, bystander activation of autoreactive T cells and rapid death of CVB-infected beta cells are also potential mechanisms that could contribute to diabetes.

#### 2.1.2. Rotavirus

Several studies investigated the role of rotavirus in the induction of autoimmunity in human and animal models, suggesting bystander induction of autoimmunity. Based on serological analysis of rotavirus antibodies and islets autoantibodies, Honeyman et al. suggested that rotavirus infection might trigger autoimmunity to pancreatic islets in high-risk children [40]. In contrast, a larger study cohort showed that rotavirus infections are unlikely to trigger beta cell autoimmunity in genetically susceptible children [41]. Studies of rhesus monkey rotavirus (RRV) infection in NOD mice indicated that RRV can induce diabetes without pancreatic infection, but it depends on the presence of insulitis with involvement of Th1 activation and proinflammatory cytokines release [42,43]. Interleukin 1 and 6, the candidate proinflammatory cytokines, are known to suppress Treg functionality allowing unchecked adaptive autoimmune response. The time of infection, age, and insulitis status seems to affect the outcomes of infection in terms of delaying, accelerating, or not affecting the onset of diabetes [43]. Moreover, rotavirus acceleration of diabetes is strain-specific, as infection of mice with RRV but not the CRW-8, which is an Australian porcine rotavirus serotype that cross react with human rotavirus serotype 3, has enhanced disease development [44]. Pane et al. proposed that diabetes acceleration by rotavirus in NOD mice occur via bystander activation: Rotavirus degraded dsRNA induce Toll-like receptor 7 (TLR7) signaling, leading to release of type-I interferon and lymphocyte activation, including autoreactive T cells, which in turn exacerbate diabetes-related autoimmunity [42]. Although TLR7 has been implicated in the bystander activation, the melanoma differentiation-associated protein 5 (MDA5), might also have a role in rotavirus-induced type-I IFN expression, and thus diabetes progression [32]. If viral-induced diabetes can occur through nonspecific mechanisms without pancreatic viral infection, e.g., proinflammatory cytokine response and lymphocyte activation, this suggests that multiple other viruses can produce similar effect [32], and hence, further studies in the field are necessary. 

### 2.2. Respiratory Infections: Influenza A Virus (IAV) as an Example

Influenza viruses primarily replicate in the respiratory tract; however, viral replication in pancreatic islets and other internal organs has also been reported and linked to bystander activation of the immune system. In 1990, Roman et al. reported that influenza virus induced insulitis and diabetes in transgenic mice expressing hemagglutinin in the pancreatic beta cells [45]. Moreover, multiple studies and case reports in the last ten years have demonstrated the possible association between influenza virus infection and diabetes development. Using both, avian strains (H7N1 and H7N3) and human strain (H1N1), Capua et al. reported that influenza viruses are able to grow in human pancreatic cell lines in vitro, whereas they lead to tissue damage and diabetes in turkeys [46]. 

Influenza viruses can reach the pancreas through viremia [47,48] and replicate in pancreatic cells from both, exocrine and endocrine origins [46]. The virus can also find its way to the pancreas through reflux from the gut to the pancreatic duct, where it finds a tolerant environment containing appropriate cell receptors and susceptible cells. Primarily, human pancreatic cells express both alpha-2,3 and alpha-2,6 sialic acid receptors, which theoretically allow both human and avian influenza strains to replicate [46]. 

Clinically, the onset of diabetes and other pancreatic diseases have been linked to influenza infections. In a case report from China in 2012, diabetic ketoacidosis was documented in a young woman after H1N1 infection [49]. In another case, a patient converted from type 2 to type 1 diabetes after influenza A virus infection during H1N1 pandemic in 2009 in Japan. This patient had a human leukocyte antigen (HLA) pattern associated with type 2 diabetes, and developed deterioration of glycemic control after the infection [50]. These cases have shed light not only on the role of viral infections, but also on the susceptibility of these patients in development of diabetes. In a study investigating type 1 diabetes onset in children hospitalized with 2009 pandemic influenza A (H1N1) virus, it was found that three children (7%) had ketoacidosis as an indication of type 1 diabetes, suggesting that H1N1 virus can be involved in disease development [51]. In addition, a recent study with a large cohort found that young children presented with respiratory infections such as influenza like illness, had increased risk of developing pancreatic islet autoimmunity [52]. 

Several studies have linked upper respiratory infections to transient increase in type-I IFN expression preceding autoimmunity/diabetes development [53]. Similarly, it was found that influenza virus stimulated IFN-α production from plasmacytoid dendritic cells (pDC), which is associated with Th1-mediated autoimmunity type 1 diabetes development [54]. Further, IAV infection induced significantly higher pDC-dependent IFN-α expression in PBMCs obtained from diabetic patients compared to those from healthy controls [54]. This increase in IFN-α expression was associated with increased numbers of pDCs, which in turn could contribute to the bystander activation of autoreactive T cells. More recently, it was reported that a highly functional CD8+ T cell response is elicited by alternative reading frame (ARF) epitopes encoded by NS1 mRNA of IAV, which might have important implications on IAV-induced autoimmunity [55]. 

Although studies have linked IAVs to diabetes, their exact role in disease prognosis and pathogenies is yet to be explored. Viral replication is thought to cause cell damage and cytokines production, similar to conditions linked to diabetes prognosis. More specifically, in situ hybridization experiments of pancreatic tissue after infection with influenza virus have indicated that viral nucleoprotein was detected in beta cells, with reduction in the number of cells staining for insulin [46]. This clearly suggests the potential effect of influenza replication on beta cells damage and insulin levels, leading to diabetes.

In the last decade, a number of studies had associated influenza infection or vaccination to type 1 narcolepsy (T1N), a chronic sleep disorder associated with autoimmune destruction of hypocretin (HCRT) neurons in the hypothalamus. This disorder is linked to the HLA-DQB1*0602/DQA1*01:02 haplotype, T cell receptors (TCR) and other immune loci, suggesting the involvement of autoimmune response in the disease pathophysiology [56,57]. However, the associated autoantigens are still not identified. Observational studied have reported an increase in T1N after the 2009 H1N1 pandemic [58]. A significantly increased risk of narcolepsy in adults and children was also linked to Pandemrix vaccination (influenza inactivated vaccine) [59,60]. Interestingly, the use of Focetria vaccination (differently formulated inactivated influenza vaccine) was not associated with increased T1N risk [61]. Considering the difference in the adjuvants as well as the antigens of both vaccines, as Pandemrix is a split-influenza vaccine while Focetria is a subunit vaccine resulting in distinct antigen formulations, this highlights the significant contribution of viral antigen composition in the development of autoimmunity [62].

Molecular mimicry of IAV antigens has been strongly suggested in T1N, in which a similarity between H1N1 specific peptides and HCRT peptides was reported. In a very recent study, Luo et al. reported that sequences of T cell receptor (TCR) α and β/complementarity-determining region (CDR) 3 were found in influenza HA nucleoprotein (NP) and HCRT tetramer-positive CD4+ T-cells, and also retrieved in INF-γ-secreting CD4+ T-cells stimulated with Pandemrix [57]. Accordingly, cross-reactivity between HCRT autoantigen and specific influenza sequences might explain how influenza infection or vaccine trigger T1N autoimmunity. Not only that, these findings also suggested the possible involvement of antigen spreading and CD8+ T-cells killing of HCRT neurons leading to this disorder, which is a known mechanism reported in other IAV-induced autoimmune disorders as well, including diabetes [63]. Nonetheless, to confirm these observations, sequence analysis of more TCRs in autoimmune responses is required.

Collectively, influenza-induced autoimmunity is explained by several mechanisms. In diabetes, the contribution of T cells bystander activation and direct beta cell damage are strongly supported. Nonetheless, in other autoimmune disorders such as T1N, molecular mimicry is believed to be the reason for disease initiation. Despite these findings, additional mechanistic and epidemiologic studies are necessary to investigate the occurrence of autoimmune disorders following influenza pandemics

### 2.3. Herpesviruses

There is an increasing evidence linking infection with herpesviruses to the development of multiple autoimmune disorders. Large epidemiological studies suggested that susceptibility to multiple sclerosis (MS) is gained in early childhood, with viral infections acting as a trigger. Consequently, herpesviruses which are a childhood infections, are considered appropriate candidates contributing to MS development [64]. Herpesviruses also persist in the host as a latent infection, and when reactivated contribute to disease pathogenesis as observed in systemic autoimmune diseases (SADs) [65,66]. 

Epstein–Barr virus (EBV) infection is suspected to have a central role in the pathogenesis of SADs [66]. In fact, high viral loads of EBV DNA were detected in the blood of systemic lupus erythematosus (SLE) patients [67,68]. Furthermore, increased EBV viral mRNAs expression was reported in SLE patients [69]. Other reports linked SLE to EBV based on serological analysis. High titers of anti-early antigen (EA) IgG and IgA were found in SLE patients compared to healthy EBV carriers [65,70]. Increased EBV viral load, high titers of EBV antibodies, and abnormal cell-mediated immunity to EBV have been also observed in rheumatoid arthritis (RA) and Sjögren’s syndrome (SS) patients [65]. These findings bear immediate relevance for the abnormal cell-mediated regulation of EBV infection and frequent virus reactivation that has been observed in RA, SS, and SLE patients [65]. 

EBV-mediated autoimmunity can also be developed through molecular mimicry, in which antibodies against EBV nuclear antigen 1 (EBNA1) cross-react with lupus associated autoantigens in SLE patients, followed by epitope spreading mechanism which will include more autoantigens [71]. Moreover, through TLR3 signaling, EBV infection induces the activation of innate immunity, and the production of type-I IFN and proinflammatory cytokines [72]. Inflammation in turn is known to contribute to autoreactivity enhancement and initiation of bystander activation [4]. Additionally, it has been found that some EBV proteins that are important in immune evasion and anti-apoptosis, such as early antigen restricted (EA/R), are homologues to cellular proteins such as *Bcl2*. Accordingly, this prevents both infected B cells and epithelial cells from apoptosis, and lead to a loss of tolerance and development of autoimmunity [66]. 

Herpesviruses are neurotropic and neurovirulent, which means they can infect cells of the central nervous system (CNS) and produce neurological illness. Of note, the eye and the CNS are immune privileged sites where the self-antigens of these organs are segregated from the adaptive immune system, in part via the blood brain barrier. Inflammation at these sites may lead to the loss of this barrier function, allowing immune cell infiltration. Herpesviruses-triggered MS can be mediated through direct lyses of CNS cells, or by the immunopathogenic host immune responses, increasing the pool of CNS specific self-antigens [64]. Several reports have indicated the presence of Herpes simplex virus (HSV) in active plaques from postmortem brain samples of MS patients [73]. In addition, it was found that the viral gene products following HSV-1 infection drive apoptosis in neuronal progenitor cells [74]. EBV, another herpesvirus that causes infectious mononucleosis (IM) has also been linked to MS [75]. Moreover, human herpesvirus 6 (HHV-6) was found to be more prevalent in MS plaques than normal MS white matter, and its reactivation has been observed in MS relapse [64]. Infection with HSV have been well-linked to herpetic stromal keratitis (HSK), which is an autoimmune corneal disease [1]. It was reported that molecular mimicry is involved in disease pathology for these viruses as well. Cornea-specific T-cell clones are found to recognize the HSV-1-derived protein UL6 in a murine model [5]. Contrarily, other studies showed that isolated T cells from HSK patients’ corneas do no cross react with UL6, suggesting that T cells may induce pathogenesis through bystander destruction in humans [76]. In addition, earlier studies reported cytomegalovirus (CMV) genome in type 1 diabetes patients [77]. Also, persistent CMV infection was found to be associated with the induction of antibodies against islet cells [78]. Further, gastrointestinal and herpes viral infections have been linked to the induction of celiac disease (CD), which is a life-long autoimmune disorder [3,79]. 

In contrast to the aforementioned studies, interestingly, some other reports have shown a protective association between herpesviruses infections and autoimmunity [3,80,81]. Serum antibodies to CMV, EBV were found to be lower in CD patients compared to healthy controls [81]. Jansen et al., found that anti-CMV, EBV, and or HSV-1 IgG levels were inversely correlated with transglutaminase type 2 antibody (TG2A) levels, suggesting a protective impact of these viruses in the pathogenesis of CD [82,83]. The protective role of infections in autoimmunity supports the “hygiene hypothesis”, which proposes that cleaner living environment leads to higher incidence of autoimmune disorders [80]. Lerner et al. found that higher CD incidences are correlated with less infectious environment [3]. Infections can abrogate autoimmunity disorders through several pathways including: post-translational modification of proteins (PTMP) form non-self to self, and therefore reducing their pathogenicity. For example, some microbial agents modify gluten and thus preventing intestine damage [3,84]. Additionally, some infections are able to shift the immune response from Th1 to Th2, establishing an immunosuppressive state in Th1-derived immune diseases, such as CD [80]. In fact, this protective effect has also been observed in parasitic infections. In mouse model, it was reported that helminths modulate the immune system and generate a Th2 environment, which protects from autoimmune diseases or relieve symptoms of established autoimmune inflammation [85]. Similarly, it was found that MS patients who are infected with helminth parasites have less severe disease compared to uninfected patients [86]. Interestingly, the administration of anti-helminthic drugs increased MS-associated disease activity [87]. Currently, the use of live helminths to treat MS and other AIDs is under clinical evaluation [88]. Other than immune system modulation towards Th2, viral infections, including infections produced by CMV and EBV, can cause apoptosis of the immune cells, thus, attenuating the immune system response, and minimizing autoimmune disease progression [3]. 

To summarize, the mechanisms by which herpesviruses trigger autoimmunity are variable. Both, molecular mimicry and bystander activation were reported in EBV- and HSV- induced autoimmunity. Not only that, EBV also has the ability to immortalize autoreactive infected B cells. In addition, as neurotropic viruses, herpesviruses can infect and kill CNS cells directly, leading to several AIDs.

### 2.4. Other Viruses

Infections with multiple other viruses such as measles and mumps (*Paramyxoviridae* family), and rubella (*Togaviridae* family), have been linked to autoimmune disorders. Specifically, mumps and rubella infections were linked to the onset of type 1 diabetes [89]. Both viruses have the ability to infect and grow in beta cells [90,91]. Further, both viruses result in CNS demyelination disease [64]. Viruses from the *Flaviviridae* family, such as Zika virus (ZIKV) and dengue virus (DENV) have been also associated to autoimmune disorders, including the recently reported ZIKV-induced Guillain–Barré syndrome and DENV-induced SLE and lupus nephritis [92,93]. Human T-lymphotropic virus type 1 (HTLV-1) from the *Retroviridae* family has been associated with CNS autoimmunity, causing myelopathy/tropical spastic paraparesis [9]. Several recent studies aimed to explain the contribution of viral infections in CNS disorders. It was suggested that antiviral immune response cross-react with human NMDA receptors (2A subunit) [94], which are responsible for excitatory glutamatergic transmission and are ion channel proteins found in nerve cells [95]. Pentapeptide similarity between the NMDA 2A receptor and viral proteins have been previously reported [96]. Using the same mechanism, other peptide commonalities with high degree of pentapeptide sharing were found between viral peptides and the human distal-less homeobox (DLX) transcription factors expressed during early fetal neurodevelopment (DLX1, DLX2, DLX5, and DLX6) [94]. Analysis of the brain-specific DLX self-antigens pentapeptide revealed matching with several viruses that have been related to neurological disorders including rubella and herpesviruses [94]. Collectively, these studies support the assumption that viral infections may relate to CNS disorders through autoimmune cross-reactions caused by molecular mimicry. 

In a recent work by Kanduc, peptide sharing analysis of five common viruses (Borna disease virus, IAV, measles, mumps, and rubella) in comparison to human proteome revealed an unexpected massive viral human peptide cross-reactivity [97]. The author explained this finding in the light of the viral eukaryogenesis hypothesis, which describes that the first eukaryotic cell had evolved from an archaeal ancestor of the eukaryotic cytoplasm, a bacterial ancestor of the mitochondria, and a viral ancestor of the nucleus. Importantly, the clinical implication of this high viral/human peptide sequence similarity confirms the significant contribution of molecular mimicry as a mechanism in viral induced autoimmunity. 

Considering the available data from epidemiological and experimental animal studies, there is a wide range of viruses that are suspected to initiate an autoimmune response, despite the lack of a clear mechanistic explanation to this phenomenon in most of the cases. Table 1 summarizes studies reporting viral induced autoimmunity in different organisms along with proposed mechanisms. Importantly, it is clear that there is no single factor responsible for triggering autoimmunity. It seems that the development of autoimmune diseases following viral infections is a multifactorial process that can be affected by different variables. Moreover, determining whether viral infection can lead to autoimmunity or protect from certain immune disorders such as diabetes [98] and CD [81], depends on multiple factors, including virus strain, genetic predisposition, host immune response, infectious dose, and time of infection [99].

## 3. Conclusions

Viral infections are a major trigger of autoimmunity. Virus-induced autoimmunity is a multi-directional process. Current data suggests that viruses can initiate autoimmunity via several pathways including molecular mimicry, epitope spreading, bystander activation and/or immortalization of infected B cells. To the contrary, a growing evidence is supporting the protective role of viruses against autoimmunity [3], where viral infections lead to the activation of regulatory immune responses, consequently suppressing the development of autoimmune reactions. This dual effect of viral infections on autoimmunity is orchestrated by different host, viral and environmental factors. Accordingly, further epidemiological and molecular research is needed to gain insights about the interplay between viral infections and host autoimmune responses, and to provide a clear mechanistic description on how a viral infection can trigger autoimmunopathies.

## Figures and Tables

**Table 1:** Examples of viral infections that have been linked to autoimmune diseases in different organisms.

Autoimmune Disease	Virus	Organism	Proposed Mechanism	Study
Acute disseminated encephalomyelitis	Influenza	Homo sapiens	Bystander activation & molecular mimicry	Sanderson et al., 2017 [100]
Autoantibodies in acquired immunodeficiency syndrome	Human Immunodeficiency virus	Homo sapiens	Bystander activation	Root-Bernstein et al., 2017 [101]
Autoimmune demyelinating disease	Semliki forest virus	Mus musculus	-	Mokhtarian et al., 2012 [102]
Autoimmune encephalitis	Herpes simplex virus	Homo sapiens	Molecular mimicry	Bradshaw et al., 2015 [103]
Autoimmune hepatitis	Esptein–Barr virus	Homo sapiens	Molecular mimicry	Cabibi et al., 2008 [104]
Autoimmune myocarditis	Coxsackie virus	Mus musculus	-	Fairweather and Rose, 2007 [105]
Autoimmune myocarditis	Coxsackievirus B3	Mus musculus	Bystander activation	Rose, 2011 [106]
Autoimmune thyroiditis	Human herpesvirus 6A (HHV-6A)	Homo sapiens	-	Caselli et al., 2017 [107]
Cryoglobulinemia	Hepatitis C virus	Homo sapiens	-	Ogishi et al., 2016 [108]
Encephalitis (Human herpes encephalitis)	Herpes simplex virus	Homo sapiens	Molecular mimicry	Armangue et al., 2014 [109]
Encephalitis and chronic neurological sequelae	Herpes simplex virus	Homo sapiens	-	Kothur et al., 2017 [110]
Encephalitis, myasthenia gravis	Japenese encephalitis virus	Mus musculus	Molecular mimicry	He et al., 2018 [111]
Experimental autoimmune encephalomyelitis	Murine Gamma-Herpesvirus 68	Mus musculus	-	Casiraghi et al., 2015 [112]
Grave’s disease	Esptein–Barr virus	Homo sapiens	-	Nagata et al., 2017 [113]
Guillain-Barré syndrome	Zika virus	Homo sapiens	Molecular mimicry	Lucchese and Kanduc, 2016 [114]
Hashimoto’s disease	Esptein–Barr virus	Homo sapiens	-	Janegova et al., 2015 [115]
Immune thrombocytopenia, autoimmune hepatitis	Hepatitis C virus	Homo sapiens	-	Tampaki and Koskinas, 2014 [116]
Encephalomyelitis	Coronavirus	Mus musculus	-	Pewe and Perlman, 2002 [117]
Induced type 1 diabetes	Encephalomyocarditis-D virus	Mus musculus	Molecular mimicry	Choi et al., 2001 [118]
Islet autoimmunity	Enteroviruses	Homo sapiens	Molecular mimicry	Honkanen et al., 2017 [119]
Lung-restricted autoimmunity	Sendai virus	Mus musculus	-	Chiu et al., 2016 [120]
Multiple sclerosis	Esptein–Barr virus	Homo sapiens	Molecular mimicry	Guan et al., 2019 [121]
Multiple sclerosis	Theiler’s virus	Homo sapiens	-	Miller et al., 2001 [122]
Multiple sclerosis	Varicella-zoster virus	Homo sapiens	-	Sotelo and Corona, 2011 [123]
Multiple sclerosis	Measles virus	Homo sapiens	-	Tucker and Andrew Paskauskas, 2008 [124]
Multiple sclerosis	Cytomegalovirus	Homo sapiens	Molecular mimicry	Vanheusden et al., 2017 [125]
Myasthenia gravis	West Nile virus	Homo sapiens/Mus musculus	Molecular mimicry	McBride et al., 2006 [126]
Myelopathy/tropical spastic paraparesis	Human T-lymphotropic virus type 1	Homo sapiens	-	Bangham et al., 2015 [127]
Polyarthritis	Hepatitis C virus	Homo sapiens	-	Zuckerman et al., 2000 [128]
Pulmonary Fibrosis	Gammaherpesvirus	Mus musculus	-	Bennion et al., 2019 [129]
Rheumatoid arthritis	Esptein–Barr virus	Homo sapiens	Epitope spreading	Dostál C et al., 1997 [130]
Rheumatoid arthritis	Cytomegalovirus	Homo sapiens	Epitope spreading	Pera et al., 2017 [131]
Pulmonary inflammation in lupus-prone mice	Influenza A virus	Mus musculus	Bystander activation & epitope spreading	Slight-Webb et al., 2015 [132]
Sjogren syndrome	Hepatitis C virus	Homo sapiens	Bystander activation	Ramos-Casals et al., 2005 [133]
Stromal keratitis	Herpes simplex virus	Homo sapiens	-	Deshpande et al., 2001 [134]
Stromal keratitis	Herpes simplex virus	Homo sapiens	-	Farooq and Shukla, 2012 [135]
Symmetric polyarthritis	Chikungunya virus	Homo sapiens	Epitope spreading	Goupil and Mores, 2016 [136]
Systemic lupus erythematosus	Cytomegalovirus	Homo sapiens	Epitope spreading	Chen et al., 2015 [137]
Systemic lupus erythematosus in porphyria cutanea tarda	Hepatitis C virus	Homo sapiens	Epitope spreading	Stölzel et al., 2002 [138]
Systemic lupus erythematous	Parvovirus B19	Homo sapiens	-	Ribeiro et al., 2015 [139]
Systemic lupus erythematous, lupus nephritis	Dengue virus	Homo sapiens	Epitope spreading	Steed and Stappenbeck, 2014 [140]
Systemic Vasculitis	Lassa Virus	Cynomolgus Macaques	-	Cashman et al., 2018 [141]
Thrombocytopenia	Hepatitis C virus	Homo sapiens	-	Dahal et al., 2017 [142]
Thyroiditis	Hepatitis C virus	Homo sapiens	Bystander activation	Ferri et al., 2017 [143]
TMEV-induced demyelinating disease	Theiler’s murine encephalomyelitis virus	Mus musculus	Molecular mimicry	Olsberg et al., 1993 [144]
Type 1 diabetes mellitus	Coxsackievirus	Homo sapiens	Molecular mimicry	Eizirik and Op de Beeck, 2018 [145]
Type 1 diabetes mellitus	Coxsackievirus B1	Homo sapiens	Molecular mimicry	Laitinen et al., 2014 [146]
Type 1 diabetes mellitus	Cytomegalovirus	Homo sapiens		Pak et al., 1988 [77]
Type 1 diabetes mellitus	Rotavirus	Mus musculus	Bystander effect	Pane et al., 2014 [41]
Type 1 diabetes mellitus	Enteroviruses	Homo sapiens/Mus musculus	-	Stene and Rewers, 2012 [147]
Vasculitis	Hepatitis C virus	Homo sapiens	-	Cacoub et al., 2014 [148]
